# Supplementary material for: Antibiotic Use Prior to COVID-19 Vaccine Is Associated with Higher Risk of COVID-19 and Adverse Outcomes: A Propensity-Scored Matched Territory-Wide Cohort
Source: Vaccines (Basel). 2023 Aug 8;11(8):1341. doi: 10.3390/vaccines11081341 (PMC10459914; doi:10.3390/vaccines11081341)
Supplement: Supplementary file 1 [file vaccines-11-01341-s001.zip › vaccines-2526342-supplementary.pdf]

Figure S1. Patient selection flow chart

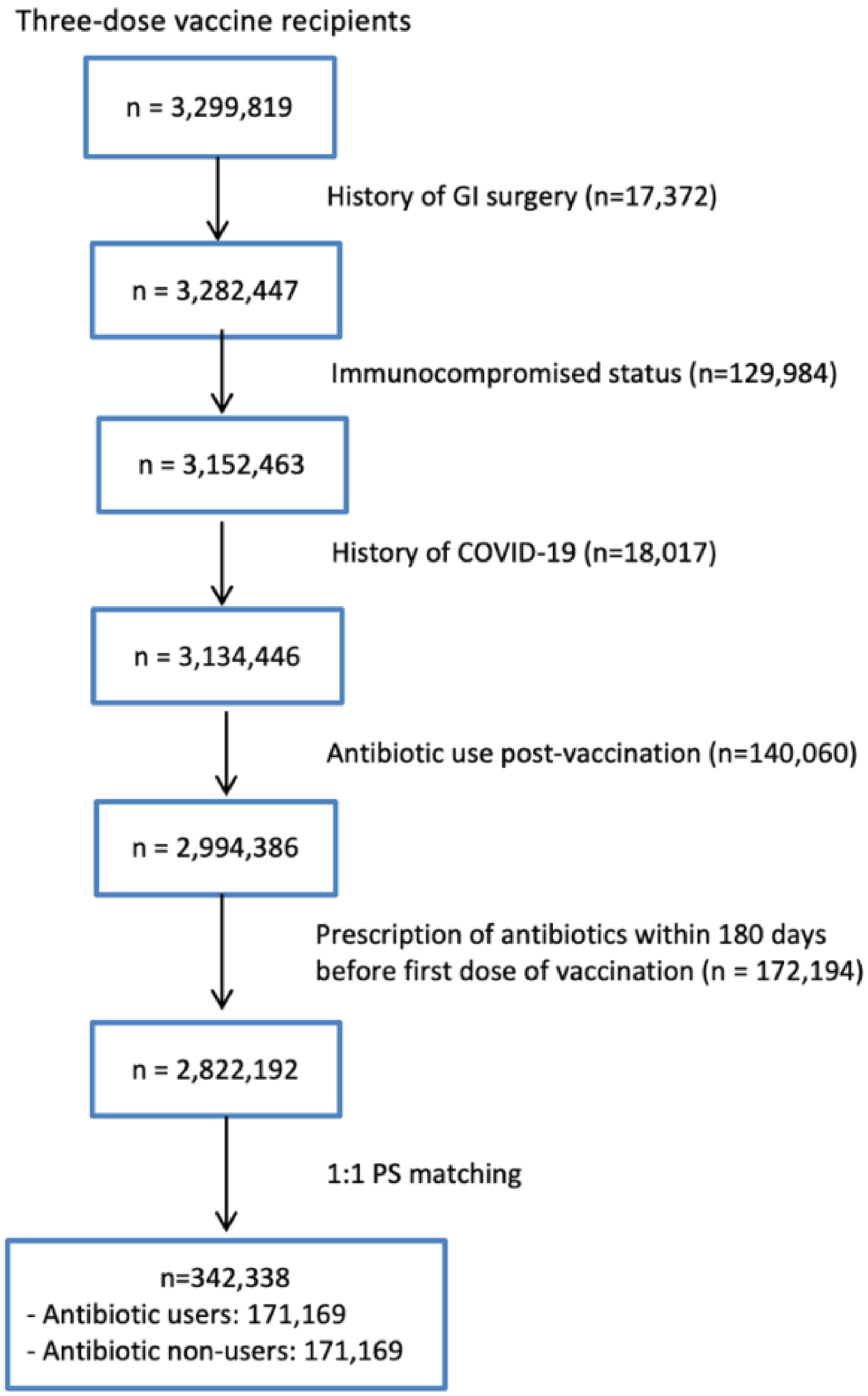

Abbreviations: GI, gastrointestinal; PS, propensity score

Table S1. Baseline characteristics of pre-vaccination antibiotic users and non-users before propensity score matching

	Pre-vaccination antibiotic non-users (n=2,822,192)	Pre-vaccination antibiotic users (n=172,194)	SMD
Age, years (mean (SD))	53.18 (17.4)	57.70 (19.2)	0.247
Sex, male (%)	1257206 (44.5)	78307 (45.5)	0.019
Charlson Comorbidity Index (mean (SD))	0.19 (0.5)	0.42 (0.8)	0.338
Vaccine platform - CoronaVac (%)	1383371 (49.0)	93930 (54.5)	0.111
Received 3rd dose (%)	1.00 (0.0)	1.00 (0.0)	<0.001
**Comorbidities – no. (%)**			
Hypertension	623388 (22.1)	51870 (30.1)	0.184
Diabetes mellitus	302934 (10.7)	26720 (15.5)	0.142
Dyslipidemia	386163 (13.7)	29597 (17.2)	0.097
Cardiovascular diseases	665681 (23.6)	58098 (33.7)	0.226
Respiratory diseases	39219 (1.4)	9924 (5.8)	0.237
Obesity diagnosis	115041 (4.1)	8697 (5.1)	0.047
Smoking	25466 (0.9)	2342 (1.4)	0.043
Alcohol use disorders	7738 (0.3)	1096 (0.6)	0.054
Ulcers	19689 (0.7)	4035 (2.3)	0.135
Moderate-severe liver disease	1218 (0.0)	388 (0.2)	0.050
Chronic renal failure	19664 (0.7)	3586 (2.1)	0.119
**Medication use in past 6 months – no. (%)**			
ACEIs	180282 (6.4)	15904 (9.2)	0.106
ARBs	202335 (7.2)	18309 (10.6)	0.122
Metformin	272156 (9.6)	21490 (12.5)	0.091
Lipid lowering agents	547298 (19.4)	46558 (27.0)	0.182
Antiplatelets	196034 (6.9)	23520 (13.7)	0.222
NSAIDs	143631 (5.1)	26609 (15.5)	0.346
Oral anticoagulants	24595 (0.9)	5056 (2.9)	0.152
Steroids	6506 (0.2)	5063 (2.9)	0.218
Antidepressants	111354 (3.9)	13145 (7.6)	0.158
Antiviral drugs	30518 (1.1)	3305 (1.9)	0.069
PPIs	187323 (6.6)	37412 (21.7)	0.443
H2RAs	280432 (9.9)	38856 (22.6)	0.347

SMD: standardized mean difference, SD: standard deviation, ACEIs: angiotensin-converting enzyme inhibitors, ARBs: angiotensin receptor blockers, NSAIDs: non-steroidal anti-inflammatory drugs, PPIs: proton pump inhibitors, H2RAs: H2 receptor antagonists

Table S2. Anti-anaerobic/anti-aerobic activity and anti-bacterial spectrum of various classes of antibiotics

Class	Route	Anti-anaerobic/ anti-aerobic activity	Anti-bacterial spectrum
Penicillins			
Benzylpenicillin	Intravenous	Anti-aerobic	Narrow spectrum
Phenoxymethylpenicillin	Oral	Anti-aerobic	Narrow spectrum
Benzathine penicillin G	Intramuscular	Anti-aerobic	Narrow spectrum
Penicillin G procaine	Intramuscular	Anti-aerobic	Narrow spectrum
Cloxacillin	Oral	Anti-aerobic	Narrow spectrum
	Intravenous	Anti-aerobic	Narrow spectrum
Flucloxacillin	Oral	Anti-aerobic	Narrow spectrum
	Intravenous	Anti-aerobic	Narrow spectrum
Amoxycillin +/- clavulanate	Oral	Anti-anaerobic	Broad spectrum
	Intravenous	Anti-anaerobic	Broad spectrum
Ampicillin +/- sulbactam	Oral	Anti-anaerobic	Broad spectrum
	Intravenous	Anti-anaerobic	Broad spectrum
Piperacillin +/- tazobactam	Oral	Anti-anaerobic	Broad spectrum
	Intravenous	Anti-anaerobic	Broad spectrum
Ticarcillin + clavulanate	Oral	Anti-anaerobic	Broad spectrum
	Intravenous	Anti-anaerobic	Broad spectrum
Cephaloporins			
Cefazolin	Intravenous	Anti-aerobic	Narrow spectrum
Cefuroxime	Oral	Anti-aerobic	Broad spectrum
	Intravenous	Anti-aerobic	Broad spectrum
Ceftibuten	Oral	Anti-aerobic	Broad spectrum
Ceftazidime	Intravenous	Anti-aerobic	Broad spectrum
Ceftriaxone	Intravenous	Anti-aerobic	Broad spectrum
	Intramuscular	Anti-aerobic	Broad spectrum
Cefotaxime	Intravenous	Anti-aerobic	Broad spectrum
Cefepime	Intravenous	Anti-aerobic	Broad spectrum
Cefaclor	Oral	Anti-anaerobic	Broad spectrum
Cefoperazone + Sulbactam	Intravenous	Anti-anaerobic	Broad spectrum
Cefoxitin	Intravenous	Anti-anaerobic	Broad spectrum
Ceftaroline	Intravenous	Anti-anaerobic	Broad spectrum
Macrolides			
Azithromycin	Oral	Anti-aerobic	Narrow spectrum
	Intravenous	Anti-aerobic	Narrow spectrum
Erythromycin	Oral	Anti-aerobic	Narrow spectrum
	Intravenous	Anti-aerobic	Narrow spectrum
Clarithromycin	Oral	Anti-aerobic	Narrow spectrum
	Intravenous	Anti-aerobic	Narrow spectrum
Carbapenems			
Imipenem + cilastatin sodium	Intravenous	Anti-anaerobic	Broad spectrum
Ertapenem	Intravenous	Anti-anaerobic	Broad spectrum
Meropenem	Intravenous	Anti-anaerobic	Broad spectrum
Monobactams			
Aztreonam	Intravenous	Anti-aerobic	Narrow spectrum
Quinolones			
Ciprofloxacin	Oral	Anti-aerobic	Broad spectrum
	Intravenous	Anti-aerobic	Broad spectrum
Levofloxacin	Oral	Anti-aerobic	Broad spectrum
	Intravenous	Anti-aerobic	Broad spectrum
Moxifloxacin	Oral	Anti-anaerobic	Broad spectrum
	Intravenous	Anti-anaerobic	Broad spectrum

Tetracyclines			
Tetracycline	Oral	Anti-aerobic	Broad spectrum
	Intravenous	Anti-aerobic	Broad spectrum
Doxycycline	Oral	Anti-aerobic	Broad spectrum
	Intravenous	Anti-aerobic	Broad spectrum
Minocycline	Oral	Anti-aerobic	Broad spectrum
	Intravenous	Anti-aerobic	Broad spectrum
Tigecyclines	Intravenous	Anti-anaerobic	Broad spectrum
Oxytetracycline	Oral	Anti-aerobic	Broad spectrum
	Intravenous	Anti-aerobic	Broad spectrum
	Intramuscular	Anti-aerobic	Broad spectrum
Aminoglycosides			
Gentamicin	Intravenous	Anti-aerobic	Broad spectrum
Amikacin	Intravenous	Anti-aerobic	Broad spectrum
Neomycin	Oral	Anti-aerobic	Broad spectrum
Tobramycin	Oral	Anti-aerobic	Broad spectrum
	Intravenous	Anti-aerobic	Broad spectrum
Streptomycin	Intravenous	Anti-aerobic	Narrow spectrum
Nitroimidazoles			
Metronidazole	Oral	Anti-anaerobic	Broad spectrum
	Intravenous	Anti-anaerobic	Broad spectrum
Tinidazole	Oral	Anti-anaerobic	Broad spectrum
Glycopeptides			
Vancomycin	Oral	Anti-aerobic	Narrow spectrum
	Intravenous	Anti-aerobic	Narrow spectrum
Teicoplanin	Intravenous	Anti-aerobic	Narrow spectrum
Others			
**Clindamycin**	Oral	Anti-anaerobic	Narrow spectrum
	Intravenous	Anti-anaerobic	Narrow spectrum
Linezolid	Oral	Anti-anaerobic	Narrow spectrum
	Intravenous	Anti-anaerobic	Narrow spectrum
Septrin	Oral	Anti-anaerobic	Broad spectrum
	Intravenous	Anti-anaerobic	Broad spectrum
Nitrofurantoin	Oral	Anti-aerobic	Narrow spectrum
Rifampicin	Oral	Anti-anaerobic	Broad spectrum
	Intravenous	Anti-anaerobic	Broad spectrum
Rifaximin	Oral	Anti-anaerobic	Broad spectrum
Daptomycin	Intravenous	Anti-aerobic	Narrow spectrum

Table S3. Association between pre-vaccination use of different classes of antibiotics and COVID-19 outcomes after vaccination with three doses of BNT162b2/CoronaVac

	Events	Person-days	No. of persons	Incidence rate (per 100,000 person-days)	Adjusted IRR (95% CI)
COVID-19					
Never users	21110	66365213	171169	31.8	-
Penicillins	20021	53458790	140883	37.5	**1.17 (1.15-1.19)**
Cephaloporins	679	1779927	5306	38.1	**1.12 (1.04-1.21)**
Macrolides	1133	3062706	7850	37.0	**1.12 (1.05-1.19)**
Carbapenems	169	325332	1179	51.9	**1.35 (1.16-1.58)**
Monobactams	0	843	3	0	-
Quinolones	2513	6559990	18024	38.3	**1.16 (1.11-1.21)**
Tetracyclines	1004	2662873	7048	37.7	**1.20 (1.13-1.28)**
Aminoglycosides	55	108485	354	50.7	**1.45 (1.11-1.89)**
Nitroimidazoles	1339	4255760	10342	31.5	1.04 (0.98-1.10)
Glycopeptides	82	183190	668	44.7	1.14 (0.92-1.42)
Others	1751	4693043	12472	37.3	**1.20 (1.14-1.26)**
COVID-19-related hospitalization					
Never users	1550	70426545	171169	2.2	-
Penicillins	2433	57145992	140883	4.3	**1.75 (1.64-1.87)**
Cephaloporins	161	1887260	5306	8.5	**2.08 (1.77-2.46)**
Macrolides	92	3279104	7850	2.8	**1.33 (1.07-1.65)**
Carbapenems	73	346055	1179	21.1	**2.65 (2.08-3.37)**
Monobactams	0	843	3	0	-
Quinolones	456	6992431	18024	6.5	**2.18 (1.96-2.42)**
Tetracyclines	160	2840762	7048	5.6	**2.04 (1.73-2.40)**
Aminoglycosides	16	116588	354	13.7	**3.36 (2.04-5.53)**
Nitroimidazoles	91	4512510	10342	2.0	**1.46 (1.18-1.8)**
Glycopeptides	40	192094	668	20.8	**2.68 (1.95-3.69)**
Others	227	5006170	12472	4.5	**1.97 (1.71-2.27)**
Severe COVID-19 (ICU admission / ventilatory support / death)					
Never users	78	70655136	171169	0.1	-
Penicillins	118	57520763	140883	0.2	**1.62 (1.22-2.16)**
Cephaloporins	6	1912331	5306	0.3	1.43 (0.62-3.31)
Macrolides	3	3294143	7850	0.1	0.96 (0.30-3.08)
Carbapenems	4	358083	1179	1.1	2.57 (0.92-7.20)
Monobactams	0	843	3	0	-
Quinolones	19	7060202	18024	0.3	**1.75 (1.06-2.91)**
Tetracyclines	8	2865757	7048	0.3	1.76 (0.84-3.70)
Aminoglycosides	1	118600	354	0.8	4.21 (0.57-31.16)
Nitroimidazoles	1	4526997	10342	0.02	0.36 (0.05-2.58)
Glycopeptides	0	198652	668	0	-
Others	8	5041373	12472	0.2	1.37 (0.66-2.84)

IRR: incidence rate ratio; CI: confidence interval
